# Patterns of healthcare contacts for decreased fetal movements in Sweden before and during the Covid-19 pandemic: a retrospective population-based cohort study

**DOI:** 10.1186/s12889-025-24812-8

**Published:** 2025-11-06

**Authors:** Anna Andrén, Ingela Rådestad, Helena Lindgren, Kerstin Erlandsson, Viktor Skokic, Anna Akselsson

**Affiliations:** 1 Department of Health Promoting Science, Sophiahemmet University, Stockholm, Sweden; 2 Department of Women’s and Children’s Health, Karolinska Institutet, Stockholm, Sweden; 3 School of Health and Welfare, Dalarna University, Falun, Sweden; 4 Department of Pelvic Cancer, Karolinska University Hospital, Stockholm, Sweden; 5 Department of Molecular Medicine and Surgery, Karolinska Institutet, Stockholm, Sweden

**Keywords:** Covid-19, Decreased fetal movements, Maternal health, Health service accessibility, Social determinants of health

## Abstract

**Background:**

The Covid-19 pandemic has posed challenges to maternal healthcare systems worldwide, impacting women’s access to and utilisation of reproductive healthcare services, particularly highlighting health inequities among minority populations. Maternal awareness of fetal movements is a measure to evaluate fetal well-being. In Sweden, women are encouraged to contact healthcare if they experience decreased fetal movements. It is unknown whether women in Sweden faced greater challenges accessing such care during the Covid-19 pandemic. The objective of this study was to examine whether the proportion of healthcare contacts for decreased fetal movements changed during the Covid-19 pandemic, and if this varied according to women’s demographic characteristics.

**Methods:**

This retrospective population-based cohort study included 18,791 who contacted healthcare due to decreased fetal movements before (1 January 2017 to 31 December 2019) and after (13 March 2020 to 31 March 2022) the onset of the Covid-19 pandemic in Region Stockholm. Women with a singleton pregnancy from gestational week 22 were included. Data were retrieved from the Swedish Pregnancy Register and the National Patient Register.

**Results:**

There was no significant difference in healthcare contacts for decreased fetal movements before compared to during the Covid-19 pandemic (17.3% vs. 17.7%). Subgroup analyses showed an increase in healthcare contacts for decreased fetal movements after the onset of the pandemic among women born in Sweden, women with a university-level education, and students. In contrast, healthcare contacts among women with BMI ≥ 30.0 kg/m² significantly declined during the pandemic period.

**Conclusions:**

Overall, contacts with healthcare due to decreased fetal movements remained consistent before and during the Covid-19 pandemic. However, variations were observed among specific subgroups, defined by BMI, region of birth, occupation, and educational level. These findings underscore the complex interplay between sociodemographic factors and maternal healthcare utilisation. To ensure equitable access to essential prenatal care during future health crises, healthcare services and communication strategies must be adapted to reflect the needs and circumstances of all demographic groups.

## Introduction

The coronavirus disease 2019 (Covid-19) pandemic significantly impacted maternal healthcare worldwide, presenting challenges to women’s access to and utilisation of reproductive and sexual health services [1]. In Sweden, studies have reported a decline in the perceived quality and accessibility of antenatal care during the pandemic [2, 3], resulting in increased stress and anxiety for women and their families [4, 5].

Maternal awareness of fetal movements is a stillbirth prevention measure and women in Sweden are encouraged to monitor fetal activity and contact their healthcare if the movements decrease in strength or frequency [6]. Findings from international studies on how the pandemic affected women’s utilisation of healthcare services for decreased fetal movements (DFM) have been inconsistent. A study from the United Kingdom reported a significant decrease in first attendances for DFM among multiparous women during the pandemic, but an increase among primiparous women [7]. In a Dutch study, 14.8% of community midwives reported referring fewer women to hospitals for DFM during the pandemic, while 64.2% of hospital-based respondents noted a decrease in consultations for this indication [8]. In Ireland, a study conducted in a large urban maternity unit found that, although the overall number of emergency department visits declined during the first lockdown, the proportion of visits due to DFM increased from 12.4% to 15.8% [9].

In the Swedish context, it remains unclear whether the Covid-19 pandemic influenced women’s care-seeking for DFM. Unlike most other countries, Sweden did not enforce a national lockdown. Instead, the public was encouraged to adhere to voluntary measures, including avoiding unnecessary travel and social gatherings. Within maternity care, infection prevention protocols led to restrictions on partner attendance during antenatal visits, ultrasound examinations, and postpartum care [10]. The use of telephone and digital consultations was expanded for non-urgent visits that could be safely conducted remotely, but the clinical management of acute complications, such as DFM, remained unchanged [11]. As vaccination rates increased and infection severity declined, restrictions were gradually lifted, and by the end of March 2022, Covid-19 was no longer classified as a notifiable disease dangerous to public health and society [12].

Examining whether women’s healthcare contacts for DFM changed during the pandemic may provide insight into whether access to essential maternal healthcare —particularly for symptoms that may indicate fetal compromise— was maintained. Understanding this is critical for improving preparedness for future health crises. Furthermore, the pandemic exposed existing vulnerabilities within the healthcare system, particularly in relation to health inequities. Individuals from ethnic minority backgrounds and those with lower socio-economic position were disproportionately affected, experiencing higher rates of severe Covid-19 infection, hospitalisation, and mortality [13–17]. Previous studies have shown that migrant women face barriers in accessing maternal healthcare services [18, 19], including consultations for DFM [20–22]. It is therefore essential to investigate whether these barriers were further exacerbated during the pandemic, potentially resulting in reduced healthcare contacts for this critical symptom of fetal distress.

Against this background, the aim of this study was to examine whether the proportion of healthcare contacts for decreased fetal movements changed during the Covid-19 pandemic, and if this varied according to women’s demographic characteristics.

## Materials and methods

We conducted a retrospective population-based cohort study encompassing women with singleton pregnancies from gestational week 22 + 0, registered at a midwifery clinic in Region Stockholm between 1 January 2017 and 31 March 2022. With 2.4 million inhabitants, Region Stockholm (as the regional public authority of Stockholm is officially known) is the most populous of Sweden’s 21 healthcare regions, spanning the city of Stockholm and its surrounding municipalities [23]. There are seven different maternity hospitals within Region Stockholm from which women can choose to give birth. In 2022, just over 26,000 children were born in the region [11].

In early 2023, data were sourced from the Swedish Pregnancy Register [24] and the National Patient Register [25]. The Swedish Pregnancy Register offers comprehensive information on each pregnancy, spanning from the first antenatal health care appointment to the postpartum visit, covering 98.4% of all births in Sweden. The National Patient Register holds data on diseases and treatments in specialised in- and outpatient care. Demographic data on the participants were obtained from the Swedish Pregnancy Register. All countries represented in the sample were divided into eight regions: Sweden (reference group); Nordic countries (except Sweden); Western Europe, North America, and Oceania; Eastern Europe, North and Central Asia; Central and South America, and Caribbean; North Africa and Middle East; Sub-Saharan Africa; and South, East and Southeast Asia. This classification, inspired by a Swedish National Board of Health and Welfare report [26], was chosen for its consideration of both the geographical location and the economic and political status of the countries. The primary outcome, ‘contact with healthcare due to DFM’, was measured using the Swedish classification of health procedures (KVÅ) code AM041 (*Examination due to DFM*) [27]. The code is used when a woman has sought care for DFM and undergoes an examination at the hospital, which typically includes a cardiotocography (CTG) assessment and, in some cases, an evaluation of fetal growth. It is not used for telephone consultations, which typically occur prior to the woman’s hospital visit.

### Statistical analysis

**Fig. 1:**
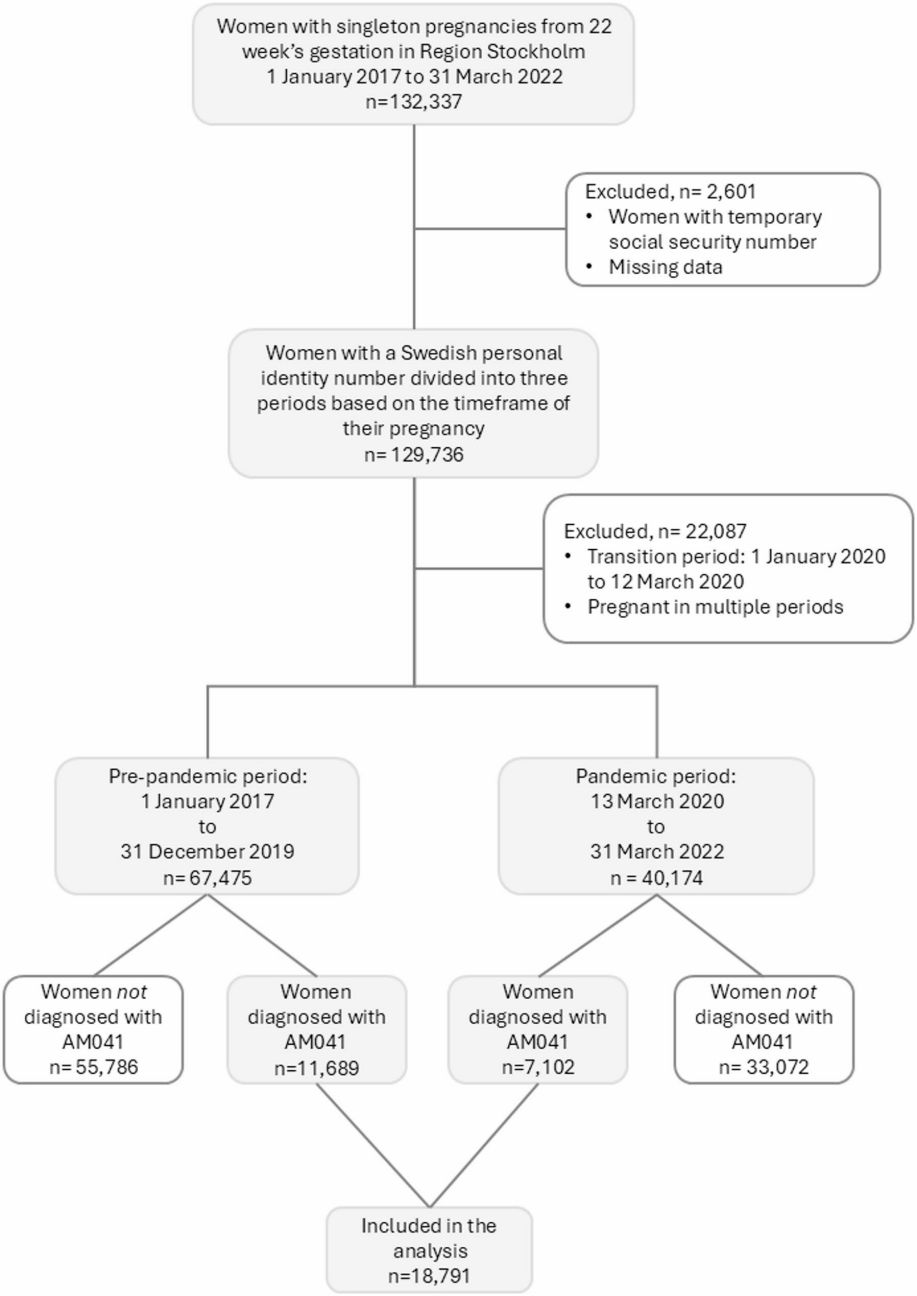
Flowchart of participant inclusion

Women without a Swedish personal identity number were excluded from the sample as the National Patient Register does not provide information on patients with a temporary social security number (*n* = 2,577). Furthermore, 24 pregnancies were excluded due to missing data regarding expected date of delivery. The data were categorised into three groups according to the woman’s gestational period. The timeframe for group one (pre-pandemic period) was specified as 1 January 2017 to 31 December 2019. Group two’s timeframe (pandemic period) spanned from 13 March 2020 to 31 March 2022. Group three, designated the transition period, encompassed 1 January 2020 to 12 March 2020. Women who were pregnant during the transition period or/and in multiple periods were excluded from the sample. Groups one and two were subsequently further divided into two subgroups: women who had received the KVÅ code AM041 and women who had not. Ultimately, 18,791 women who had received AM041 during pregnancy, were included in the analysis (Fig. 1).

To evaluate differences in the distribution of variables of interest between the two time periods, we applied the chi-square test. As a measure of association, we calculated percentage ratios, which are presented as risk ratio (RR) or adjusted risk ratio (aRR). To account for potential confounding factors, we used a log-binomial regression model to adjust the RR and calculate 95% confidence intervals (CI). The confounding factors considered in this analysis included maternal age and parity. Additionally, we stratified the sample based on the demographic characteristics parity, maternal age, birth region, educational level, occupation, body mass index (BMI), family situation, and medical history. All analyses were conducted at a pregnancy level and do not account for clustering in the data, as the same woman may have been pregnant multiple times during the study period. Statistical analyses were performed using the R statistical software (version 4.3.2).

## Results

Descriptive demographic characteristics of the participants in the sample is presented in Table 1. We found no statistically significant difference in the proportion of contacts with healthcare due to DFM before compared to after the onset of the Covid-19 pandemic (17.3% vs. 17.7%, aRR 1.02, CI 0.99–1.05). We observed that certain subgroups showed slight variations, which may indicate potential patterns of change. Specifically, for women with BMI of 30.0 or above, the proportion of contacts with healthcare for DFM declined during the pandemic period compared to the pre-pandemic period (22.5% vs. 20.5%, aRR 0.92, CI 0.86–0.98). In contrast, among women born in Sweden, a small increase in healthcare contacts for DFM was observed during the pandemic period, with an aRR of 1.04 (CI 1.01–1.07). Similarly, modest increases were seen among women with a university-level education (aRR 1.06, CI 1.03–1.10) and among students (aRR 1.10, CI 1.01–1.21).

**Table 1:** Comparison of demographic characteristics among women with Singleton pregnancies from 22 weeks’ gestation in region Stockholm contacting healthcare for DFM from 1 January 2017 to 31 December 2019 (pre-pandemic period) versus 13 March 2020 to 31 March 2022 (pandemic period)

Demographic characteristics	Pre-pandemic period *n*/*N* (%)	Pandemic period *n*/*N* (%)	RR (95% CI)	aRR (95% CI)^a^
Total number	11,689/67,475 (**17.3**)	7102/40,174 (**17.7**)	1.02 (0.99–1.05)	1.02 (0.99–1.05)
Parity				
Primipara	6638/30,693 (**21.6**)	4056/18,654 (**21.7**)	1.01 (0.97–1.04)	1.01 (0.98–1.05)^b^
Multipara	5051/36,782 (**13.7**)	3046/21,520 (**14.2**)	1.03 (0.99–1.07)	1.03 (0.99–1.08)^b^
Maternal age				
≤ 24 years	1086/4250 (**25.6**)	521/2032 (**25.6**)	1.00 (0.92–1.10)	1.00 (0.92–1.09)^c^
25–34 years	7624/43,119 (**17.7**)	4741/25,929 (**18.3**)	1.03 (1.00–1.07)	1.03 (1.00–1.06)^c^
≥35 years	2979/20,106 (**14.8**)	1840/12,213 (**15.1**)	1.02 (0.96–1.07)	1.01 (0.95–1.06)^c^
Birth region				
Sweden	7588/42,350 (**17.9**)	4633/24,984 (**18.5**)	1.03 (1.00–1.07)	1.04 (1.01–1.07)*
Nordic countries (except Sweden)	145/825 (**17.6**)	71/476 (**14.9**)	0.85 (0.65–1.10)	0.84 (0.65–1.08)
Western Europe, North America, and Oceania	241/1807 (**13.3**)	180/1189 (**15.1**)	1.14 (0.95–1.36)	1.13 (0.94–1.35)
Eastern Europe, North and Central Asia	653/4432 (**14.7**)	364/2653 (**13.7**)	0.93 (0.83–1.05)	0.93 (0.83–1.05)
Central & South America, and Caribbean	209/1102 (**19.0**)	121/624 (**19.4**)	1.02 (0.84–1.25)	1.02 (0.83–1.25)
North Africa and Middle East	1083/5612 (**19.3**)	675/3319 (**20.3**)	1.05 (0.97–1.15)	1.04 (0.96–1.13)
Sub-Saharan Africa	422/3110 (**13.6**)	256/1958 (**13.1**)	0.96 (0.83–1.11)	0.96 (0.83–1.11)
South, East and Southeast Asia	589/3780 (**15.6**)	420/2601 (**16.1**)	1.04 (0.92–1.16)	1.06 (0.95–1.19)
Other	14/69 (**20.3**)	5/32 (**15.6**)	0.77 (0.30–1.95)	
Missing	745/4388 (**17.0**)	377/2338 (**16.1**)		
Educational level				
University	6456/39,060 (**16.5**)	4379/24,785 (**17.7**)	1.07 (1.03–1.11)	1.06 (1.03–1.10)**
High school	3167/16,058 (**19.7**)	1747/9236 (**18.9**)	0.96 (0.91–1.01)	0.97 (0.92–1.02)
Elementary school	476/2680 (**17.8**)	236/1405 (**16.8**)	0.95 (0.82–1.09)	0.98 (0.85–1.13)
Shorter than 9 years	112/896 (**12.5**)	41/437 (**9.4**)	0.75 (0.53–1.05)	0.77 (0.55–1.08)
Missing	1478/8781 (**16.8**)	699/4311 (**16.2**)		
Occupation				
Employed	8574/49,045 (**17.5**)	5229/29,017 (**18.0**)	1.03 (1.00–1.06)	1.03 (1.00–1.06)
Student	928/5219 (**17.8**)	641/3314 (**19.3**)	1.09 (0.99–1.19)	1.10 (1.01–1.21)*
Parental leave	518/3859 (**13.4**)	296/2283 (**13.0**)	0.97 (0.85–1.10)	0.97 (0.85–1.11)
Sickness abscent	231/851 (**27.1**)	117/462 (**25.3**)	0.93 (0.77–1.13)	0.94 (0.78–1.13)
Unemployed	302/1559 (**19.4**)	233/1337 (**17.4**)	0.90 (0.77–1.05)	0.91 (0.78–1.06)
Other	322/2181 (**14.8**)	179/1260 (**14.2**)	0.96 (0.81–1.14)	0.98 (0.83–1.16)
Missing	814/4761 (**17.1**)	407/2501 (**16.3**)		
Body mass index				
≤ 18,4 kg/m²	270/1741 (**15.5**)	181/1002 (**18.1**)	1.16 (0.98–1.38)	1.17 (0.99–1.39)
18.5–24,9 kg/m²	6336/39,743 (**15.9**)	3756/22,845 (**16.4**)	1.03 (0.99–1.07)	1.03 (0.99–1.07)
25.0–29,9 kg/m²	2867/15,610 (**18.4**)	1923/10,144 (**19.0**)	1.03 (0.98–1.09)	1.03 (0.98–1.08)
≥ 30,0 kg/m²	1677/7458 (**22.5**)	1054/5134 (**20.5**)	0.91 (0.85–0.98)	0.92 (0.86–0.98)*
Missing	539/2923 (**18.4**)	188/1049 (**17.9**)		
Family situation				
Cohabiting with the baby’s father	10,622/61,969 (**17.1**)	6487/36,900 (**17.6**)	1.03 (1.00–1.05)	1.03 (1.00–1.05)
Single parent	242/1254 (**19.3**)	183/888 (**20.6**)	1.07 (0.90–1.27)	1.08 (0.91–1.27)
Other family situation	489/2521 (**19.4**)	232/1381 (**16.8**)	0.87 (0.75–1.00)	0.88 (0.77–1.02)
Missing	336/1731 (**19.4**)	200/1005 (**19.9**)		
Medical history				
Stillbirth	108/403 (**26.8**)	66/233 (**28.3**)	1.06 (0.81–1.37)	1.03 (0.79–1.33)
Mental illness	2315/10,472 (**22.1**)	1779/8123 (**21.9**)	0.99 (0.94–1.05)	0.99 (0.94–1.05)
Medical and/or psychologic treatment for mental illness before and/or during pregnancy	937/4048 (**23.1**)	841/3674 (**22.9**)	0.99 (0.91–1.07)	0.98 (0.91–1.06)
Tobacco/snuff use at registration at the midwifery clinic	478/2290 (**20.9**)	274/1248 (**22.0**)	1.05 (0.92–1.20)	1.06 (0.93–1.20)

^a^Adjusted for maternal age and parity

^b^Adjusted for maternal age

^c^Adjusted for parity

*P-value < 0.05

**P-value < 0.001

When women born outside Sweden were stratified into one group and compared with Swedish-born women, the proportion of healthcare contacts for DFM was lower in both time periods: 16.2% versus 17.9% during the pre-pandemic period, and 16.3% versus 18.5% during the pandemic period. We found no differences in birth outcomes among all women with a singleton pregnancy giving birth from gestational week 22 between the two time periods (Table 2 ).

**Table 2:** Mode of birth and birth outcomes among 107,649 women with a singleton pregnancy giving birth in Region Stockholm from 22 weeks’ gestation from 1 January 2017 to 31 December 2019 (pre-pandemic period) and from 13 March 2020 to 31 March 2022 (pandemic period)

Mode of birth and neonatal outcomes	Pre-pandemic period(n=67,475)n (%)	Pandemic period(n=40,174)n (%)
Spontaneous start of labour	46,858 (69.4)	26,263 (65.4)
Labour induction	13,576 (20.1)	9,921 (24.7)
Spontaneous vaginal birth	50,453 (74.8)	29,797 (74.2)
Vacuum Extraction	3,365 (5.0)	2,124 (5.3)
Elective cesarean section	7,063 (10.5)	4,002 (10.0)
Emergency cesarean section	6,576 (9.7)	4,241 (10.5)
Birth gestation <37+0	3,085 (4.6)	1,856 (4.6)
Birth gestation >41+6	3,378 (5.0)	1,204 (3.0)
Birthweight <2 SD	2,227 (3.3)	1,270 (3.2)
Birthweight <10^th^ centile	7,124 (10.6%)	4,100 (10.2%)
Apgar score 10 at 5 minutes	58,049 (86.3)	3,4701 (86.6)
Apgar score <10at 5 minutes	9,232 (13.7)	5,351 (13.4)
Apgar score <7 at 5 minutes	890 (1.3)	570 (1.4)
Apgar score <4at 5 minutes	335 (0.5)	196 (0.5)
Admission to neonatal intensive care unit	4,537 (6.7)	2,567 (6.4)
Stillbirth	195 (0.3)	114 (0.3)

## Discussion

This large population-based retrospective cohort study conducted in Region Stockholm examined if the proportion of healthcare contacts due to DFM changed during the Covid-19 pandemic, and if this varied according to women’s demographic characteristics. Overall, we found no statistically significant difference between the two time periods. However, we observed a change in healthcare contacts among certain subgroups related to BMI, birth region, occupation, and educational level.

We found that, in Region Stockholm, 17.3% of women with singleton pregnancies from 22 weeks’ gestation contacted healthcare due to DFM during the pre-pandemic period, compared to 17.7% during the pandemic period. These findings contrast with international studies that found a change in healthcare-seeking behaviour for DFM during the pandemic compared to before [7–9]. Unlike our study, these studies focused solely on the first wave of the pandemic. It is possible that during this time, the pandemic had a greater impact on healthcare contacts for this complication, as measures to reduce virus transmission may have been stricter. Given the vulnerability of pregnant women during the pandemic, one might have expected an increased need to seek care for complications such as DFM. Thus, the consistency in healthcare contacts observed across these time periods may reflect the robustness of the Swedish maternity healthcare system. A general trend during the pandemic in Sweden was that patients refrained from seeking medical care to avoid infection, prevent transmission to others, or reduce strain on healthcare systems [28]. However, similar to the findings from our study, maternal healthcare services proved to be an exception to this pattern, with women continuing to utilise prenatal and postnatal care services at consistent levels [28, 29]. Furthermore, our findings may suggest that midwives, despite the challenging circumstances of the pandemic [30, 31], effectively communicated the importance of monitoring fetal movements and seeking care in case of decreased fetal activity. National guidelines from the Swedish National Board of Health and Welfare [6] emphasise the importance of fetal movement awareness as a preventive measure against stillbirth, likely prompting midwives to prioritise this information. Our findings underscore the need for country-specific analyses, as pandemic responses and their impacts vary globally, even among high-resource countries.

The consistency in healthcare contacts for DFM that we observed before and after the onset of the Covid-19 pandemic may be explained by several interrelated factors. Early in the pandemic, pregnant women were identified as a high-risk group for severe Covid-19, and infection with severe acute respiratory syndrome coronavirus 2 (SARS-CoV-2) at the time of birth has been associated with increased rates of adverse pregnancy and birth outcomes [32, 33]. Awareness of these risks may have made some women more inclined to seek hospital care when concerned about complications. A history of mental illness, such as depression or anxiety, has previously been identified as a predictor of healthcare contact due to DFM [20], which was confirmed in the findings of this study (Table 1). While some women may have been hesitant to seek hospital care due to fear of Covid-19 exposure [34], others may have become more vigilant about fetal movements as a result of increased anxiety during the pandemic [35].

We found a statistically significant reduction in contacts with healthcare due to DFM among women with BMI of 30.0 or above during the pandemic period compared to the pre-pandemic period (22.5% versus 20.5%). This decline in healthcare contacts among a recognised high-risk group [36–38] is particularly concerning, as it may lead to missed opportunities for early detection and intervention in fetal health issues. This finding also reflects existing health disparities within Swedish society, as overweight and obesity are more common among migrant women and among women with low educational level [39, 40]. Women with obesity, who are at greater risk for severe Covid-19 complications [41], may have been more reluctant to visit healthcare facilities during the pandemic period [42]. Conversely, the additional monitoring and examinations routinely provided to women with elevated BMI [43] might paradoxically contribute to reduced healthcare-seeking behaviour, possibly due to a perception that sufficient care was already being provided. Given the heightened health risks associated with obesity in pregnancy, these checks may have become even more comprehensive during the pandemic. The intensified precautions and increased attention to high-risk individuals during the Covid-19 crisis [44] may have resulted in more thorough follow-up for this group, reflecting the resilience and adaptability of the Swedish maternal healthcare system.

Our subgroup analysis revealed that contacts with healthcare due to DFM increased among women born in Sweden, those with university-level education, and students. During the pandemic, when in-person healthcare visits were restricted, access to reliable online health information became essential. As noted by maternity care providers, the closure of certain services and the transition to virtual or telephone-based care appointments had a greater negative impact on disadvantaged or vulnerable groups in society [34]. It is possible that Swedish-born women, women with university education, and students were able to navigate online health resources more effectively, adapt to utilising telehealth services, access online consultations, and understand information about health guidelines and recommendations [22, 45, 46]. Higher educational attainment is associated with a more favourable socio-economic position, which may facilitate easier access to healthcare services [47]. Overall, the combination of digital literacy, health awareness, and adaptability to online platforms may have played a role in the increased proportion of healthcare contacts observed among these specific groups. Although the differences we observed are small with limited clinical relevance, our findings may indicate that the Covid-19 pandemic exacerbated existing inequities in maternity care in Sweden.

A lower proportion of healthcare contacts for DFM was also observed among women born outside Sweden, compared to Swedish-born women, across both the pre-pandemic and pandemic periods. This finding suggests a persistent disparity that cannot be attributed solely to the circumstances of the Covid-19 pandemic. Instead, it may reflect the challenges migrant face accessing health-related information in general, and information about DFM in particular [21, 22, 48]. Policymakers should consider this disparity when developing maternal healthcare policies and guidelines, and ensure that resources are allocated to promote equitable access to care for all women.

### Strengths and limitations

To our knowledge, this is the first Swedish study to examine the potential impact of the Covid-19 pandemic on women’s healthcare contacts for DFM, and to explore whether this impact varied according to women’s demographic characteristics. A strength of the study is its large sample size and the use of comprehensive register-based data, which allowed us to include the majority of women with singleton pregnancies in Region Stockholm during the study periods. Due to its large population and the presence of multiple maternity hospitals, the organisation of maternity care in Region Stockholm differs from that in other regions of Sweden. As a result, the generalisability of our findings to other healthcare regions may be limited. Nevertheless, we believe the study provides some valuable insights for national policymakers in preparing for future pandemics or other public health crises.

There are methodological limitations to this study. The KVÅ code AM041 is applied to examinations for DFM that do not result in further interventions and is not used for consultations that lead to inpatient admission. Therefore, it is reasonable to assume that the actual proportion of healthcare contacts for DFM in the study setting is higher than our findings suggest. Additionally, this code does not reflect how many women experienced DFM during this period, but instead how many women received hospital care for this complication. Moreover, variations in the application of this code among healthcare providers may potentially compromise the validity of the study’s findings. Due to limitations in the registers used, we were unable to include women with temporary social security numbers, such as immigrants, refugees, undocumented migrants, and international students temporarily residing in the country. Consequently, the study does not fully represent the diverse population of women experiencing DFM, potentially leading to bias in the findings. We did not conduct any specific time analyses and therefore cannot comment on whether healthcare contacts for DFM varied during the different waves of the pandemic. These analyses might have provided information that would have contributed to a greater understanding of how aspect such as the level of infection spread and public health recommendations affected women’s healthcare-seeking behaviour for DFM.

## Conclusions

This study found that, overall, healthcare contacts for DFM remained consistent among pregnant women in Region Stockholm before and during the Covid-19 pandemic, suggesting a degree of resilience within the Swedish maternity healthcare system. However, variations observed among specific subgroups point to potentially unequal impacts of the pandemic across different population groups. These differences —related to factors such as BMI, birth region, educational level, and occupation— may reflect underlying disparities in healthcare access. Notably, Swedish-born women, women with university-level education, and students showed a modest increase in healthcare contacts for DFM during the pandemic, while women with elevated BMI showed a slight decline. Women born outside Sweden consistently had lower proportions of healthcare contacts in both periods, indicating a persistent disparity for this group of women. The study highlights the complex interplay between sociodemographic factors and maternal healthcare utilisation. It underscores the importance of addressing structural barriers to care, particularly among high-risk and migrant populations. To promote equitable access to essential maternal healthcare during future pandemics or public health crises, it is crucial that healthcare services and communication strategies are adapted to meet the diverse needs and circumstances of all demographic groups.

## Data Availability

No datasets were generated or analysed during the current study.

## Abbreviations

aRR: Adjusted Risk Ratio
BMI: Body Mass Index
CI: Confidence Interval
Covid-19: Coronavirus Disease 2019
CTG: Cardiotocography
DFM: Decreased Fetal Movements
KVÅ: Classification of Health Care Procedures
RR: Risk Ratio
SARS-CoV-2: Severe Acute Respiratory Syndrome Coronavirus 2
